# Closed-Loop Brain Stimulation

**DOI:** 10.1016/j.biopsych.2023.09.014

**Published:** 2024-03-15

**Authors:** Christoph Zrenner, Ulf Ziemann

**Affiliations:** a Temerty Centre for Therapeutic Brain Intervention, Centre for Addiction and Mental Health, Toronto, Ontario, Canada; b Department of Psychiatry, University of Toronto, Toronto, Ontario, Canada; c Institute for Biomedical Engineering, University of Toronto, Toronto, Ontario, Canada; d Department of Neurology & Stroke, University of Tübingen, Tübingen, Germany; e Hertie Institute for Clinical Brain Research, University of Tübingen, Tübingen, Germany

**Keywords:** Brain state, Closed-loop stimulation, EEG, Real-time, TMS

## Abstract

In the same way that beauty lies in the eye of the beholder, what a stimulus does to the brain is determined not simply by the nature of the stimulus but by the nature of the brain that is receiving the stimulus at that instant in time. Over the past decades, therapeutic brain stimulation has typically applied open-loop fixed protocols and has largely ignored this principle. Only recent neurotechnological advancements have enabled us to predict the nature of the brain (i.e., the electrophysiological brain state in the next instance in time) with sufficient temporal precision in the range of milliseconds using feedforward algorithms applied to electroencephalography time-series data. This allows stimulation exclusively whenever the targeted brain area is in a prespecified excitability or connectivity state. Preclinical studies have shown that repetitive stimulation during a particular brain state (e.g., high-excitability state), but not during other states, results in lasting modification (e.g., long-term potentiation) of the stimulated circuits. Here, we survey the evidence that this is also possible at the systems level of the human cortex using electroencephalography-informed transcranial magnetic stimulation. We critically discuss opportunities and difficulties in developing brain state–dependent stimulation for more effective long-term modification of pathological brain networks (e.g., in major depressive disorder) than is achievable with conventional fixed protocols. The same real-time electroencephalography-informed transcranial magnetic stimulation technology will allow closing of the loop by recording the effects of stimulation. This information may enable stimulation protocol adaptation that maximizes treatment response. This way, brain states control brain stimulation, thereby introducing a paradigm shift from open-loop to closed-loop stimulation.

Transcranial magnetic stimulation (TMS) is established by now as a U.S. Food and Drug Administration–approved treatment for a variety of psychiatric disorders, e.g., major depressive disorder (MDD) (1, 2, 3) and obsessive-compulsive disorder (4), and for smoking cessation (5). However, the overall effect sizes are only moderate, and a substantial percentage of patients (typically 50%–90%) are nonresponders (1, 2, 3,6). Response rate may be increased by several strategies, such as using accelerated stimulation protocols combined with individual cortical targeting (7) according to magnetic resonance imaging–based connectomics (8,9). However, the efficacy of these strategies has been variable across trials, and in spite of strong evidence that personalized spatial targeting improves outcome, the clinical effect of the current approach is limited (10).

Another strategy for enhancing therapeutic efficacy may be individualization of the timing of TMS by synchronizing stimulation with specific instantaneous electrophysiological brain states. The response of the brain to the same repeated stimulus is highly variable and strongly depends on the ongoing activity of the stimulated cortex at the time of stimulation (11, 12, 13, 14). For example, single-pulse TMS of the primary motor cortex results in larger motor evoked potentials (MEPs) if applied during the trough rather than the peak of the ongoing local sensorimotor mu rhythm in the resting-state electroencephalography (EEG) (15). This demonstrates that different phases of a brain oscillation represent different excitability states to external stimulation. In addition to phase of EEG oscillations, power (16,17), phase synchronization between nodes in the motor network (18), and spatiotemporally distributed compound phase-pattern EEG information (19) contribute significantly to excitability states in the motor cortex.

These observations lead to the fundamentally important question of whether repeated stimulation during instants of a specific brain excitability or connectivity state would induce long-term change in excitability or connectivity more effectively than random stimulation uninformed of the ongoing brain state (20). Previous experiments with rat hippocampal slice preparations have demonstrated that a single high-frequency (100 Hz) quadruplet pulse resulted in long-term potentiation (LTP) only when applied during the peak of the ongoing theta rhythm as measured by local field potentials, while, in contrast, the identical stimulation led to long-term depression (LTD) of previously potentiated synapses when applied during the trough of the theta rhythm (21,22).

Synaptic plasticity in the form of LTP and LTD is crucial for fundamental neurobiological processes such as memory formation and learning (23,24). Many psychiatric diseases are considered to be brain network disorders. For example, people with MDD have shown abnormal functional resting-state connectivity in functional magnetic resonance imaging studies in the default mode network, central executive network, and salience network (25,26). TMS of the primary target in MDD, the dorsolateral prefrontal cortex (DLPFC), may exert its antidepressive effects by normalizing these altered functional connectivities (27,28), probably through induction of synaptic plasticity in these networks (27,29,30).

Here, we elucidate the possibility and provide first evidence that repetitive TMS (rTMS) synchronized with specific brain states induces LTP-/LTD-like plasticity in the human cortex more efficiently than open-loop TMS uninformed of brain state. We also introduce the concept of closed-loop stimulation approaches as an emergent principle of highly individualized therapeutic brain stimulation. The methodology of EEG-synchronized TMS is illustrated in Figure 1.

**Figure 1:**
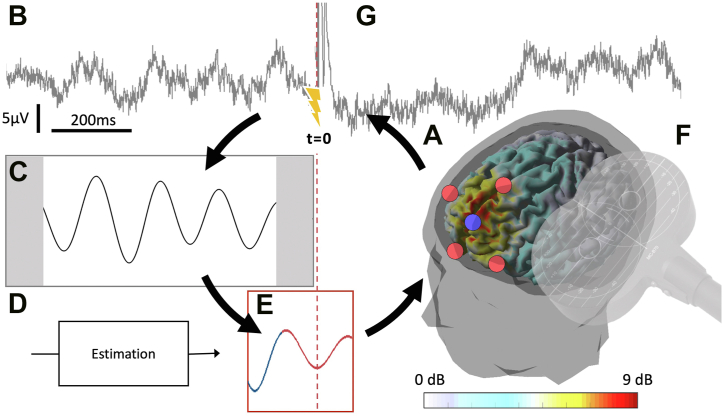
Closed-loop stimulation with electroencephalography (EEG)-informed transcranial magnetic stimulation. **(A)** The midfrontal theta oscillation is extracted using a 5-channel Surface Laplacian EEG montage (color bar indicates average theta oscillation signal-to-noise ratio, localized to cortical sources). **(B)** Overlapping windows of ongoing EEG data are acquired in real-time. **(C)** The signal is filtered in the theta band, leading to artifacts at the edge (gray shading). **(D)** Instantaneous phase is estimated using a forward prediction algorithm, taking into account signal delays. **(E)** The predefined target phase (here, the trough of the oscillation) is detected. **(F)** Stimulation is applied synchronized to the detected state. **(G)** Outcome markers of target network modulation may be extracted from a running average of transcranial magnetic stimulation–evoked EEG responses, which can then be used in a slower time scale second-order loop to update the parameters of subsequent stimulation, including the target state defined in **(E)**.

Midfrontal theta oscillation signal-to-noise ratio data reproduced with permission from Gordon *et al.* (70).

## Fundamental Concepts of Closed-Loop Stimulation

**Figure 2:**
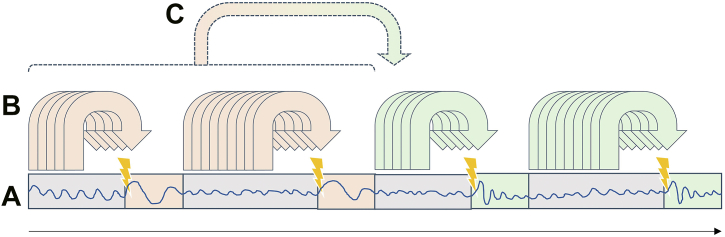
Brain signal readout processing. **(A)** Brain signal (blue oscillation) consisting of ongoing (gray shading) and stimulus-evoked (colored shading) areas. **(B)** Ongoing brain signals determine whether or not a given stimulus is applied at that moment based on a preceding window of data, termed “trigger function.” **(C)** Stimulus parameters and trigger conditions are periodically reconfigured depending on a brain readout that may be different from the readout used for **(B)**, termed “update function.”

Closed-loop stimulation is characterized by a bidirectional interaction between the therapy system and the brain, with specific parameters of a given stimulus affecting brain dynamics and a simultaneous neurophysiological readout from the brain being used to adjust the parameters of the (subsequent) stimulus. In general, the brain readout can perform 2 different roles on different time scales, a first-order trigger function and a second-order update function. The trigger function continuously determines whether and with which parameters to stimulate at that moment (i.e., depending on the occurrence and magnitude of events detected in the brain signal). This requires a real-time analysis of the ongoing signal with a temporal resolution that depends on the specific application (i.e., milliseconds if the stimulus timing depends on oscillatory phase or seconds if the stimulus amplitude depends on spectral power). In contrast, the update function evaluates whether the current settings of the trigger functions are appropriate to achieve the desired neuroplastic effect. It uses data on a longer time scale of at least minutes (including evoked responses and potentially additional measures including behavioral metrics) to continuously determine whether the intervention is working as currently configured or whether the trigger parameters need to be adjusted. See Figure 2 for an illustration of these 2 functions of the brain signal readout.

Such a scenario is comparatively straightforward if the action of the stimulation on the brain can be considered to be instantaneous, invariant over time, without aftereffects, and largely independent of ongoing brain dynamics. This is the idealized case in prosthetic brain implants that seek to up- or downregulate abnormally low or high brain activity occurring at a given moment in time, such as deep brain stimulation (DBS) in response to dyskinesia (31) or seizure events (32). In that scenario, the readout from the brain is an estimator of the presence and severity of dysfunctional activity. The trigger function can be implemented as a simple control system that achieves a set point (such as using a thermostat and a radiator for temperature control) or a switch that turns DBS on or off in response to a threshold (e.g., spectral band power) (33). The second-order update function is typically performed by the treating physician, with the positioning of the electrode during surgery informed by the electrophysiological spectral signature and subsequent changes in the stimulation parameters of the DBS additionally by therapeutic efficacy. It is possible to automate such a DBS parameter optimization algorithm (34), in which case the human operator performs a third-order configuration function.

However, the scenario that is being discussed here is different. We consider the application of closed-loop stimulation with EEG and TMS that is applied as an intermittent treatment intervention, typically for less than an hour per day over a period of weeks. The goal is to support the therapeutic process by which brain dynamics shift from a pathological state back toward a physiological state. Brain stimulation is used not to achieve an instantaneous, simple up- or downregulation of circuit activity, but rather to induce a specific neuromodulatory change, e.g., through long-term synaptic plasticity. The fact that neuroplastic changes induced by TMS (and also the therapeutic effects) take time to manifest themselves complicates the application of closed-loop therapeutic interventions in several ways. Firstly, it is no longer possible to immediately determine from the recorded brain signal whether the intervention is effective because the consequences of any changes to the parameters can only be assessed after the synaptic plastic changes have occurred. Secondly, it is not obvious which EEG-derived brain state to synchronize the stimulation with. Instead of quantifying and responding to direct measures of symptom severity, we now need to identify a readout that detects brain states during which TMS is more effective at inducing (over a period of time) the desired synaptic plastic change.

Based on these considerations, we propose the following 4 basic measures to characterize a closed-loop therapy approach: 1) the degrees of freedom of the stimulation system (on/off, intensity, current waveform, location, orientation, etc.); 2) the number of parameters extracted from the EEG signal, their temporal resolution, and the accuracy of derived brain state estimates (oscillatory phase, spectral amplitude, connectivity, etc.); 3) the temporal resolution of the first-order trigger function (milliseconds to seconds); and 4) the refresh rate of any second-order update function (minutes to days) that evaluates whether the desired outcome is being achieved and adjusts the stimulation configuration accordingly.

**Figure 3:**
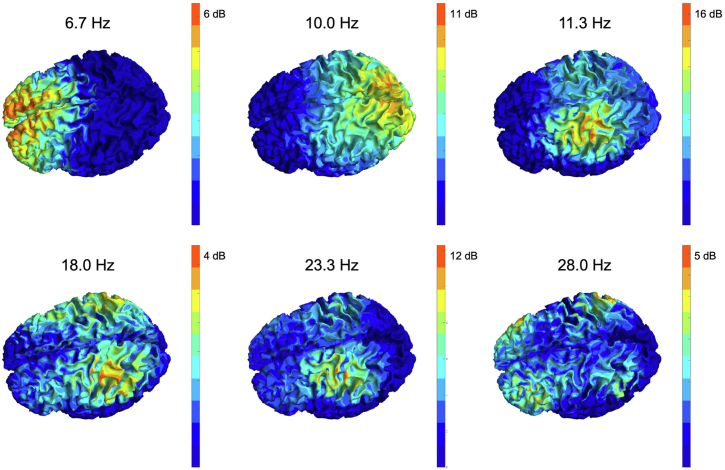
Spatial distribution and signal-to-noise ratio of different brain oscillations in a single representative electroencephalography dataset. Spectra were estimated from 10 minutes of high-density electroencephalography data recorded at rest with eyes open. The periodic component of the spectrum was estimated by subtracting the aperiodic 1/f component of the spectrum. The resulting relative power of the periodic component represents signal-to-noise ratio and can be expressed in units of decibels (dB). The resulting data at 6 different frequencies corresponding to peaks in the average spectrum of this individual was projected to the cortical source space using the linearly constrained minimum variance beamforming method applied to the individual head model. 6.7 Hz: midfrontal theta; 10.0 Hz: occipital (visual) alpha; 11.3 Hz: sensorimotor alpha; 18.0 Hz: sensorimotor beta; 23.3 Hz: sensorimotor alpha first harmonic; 28.0 Hz: frontoparietal beta. Unpublished data collected and analyzed by the authors and reproduced for illustrative purposes.

We will discuss current challenges in implementing effective autonomous second-order update functions at the end of this review. With regard to the first-order trigger function, we will discuss the potential of using the oscillatory phase as a temporal target for the optimized induction of plasticity. This approach is motivated by the fact that scalp potentials measured in the EEG are predominantly generated by postsynaptic potentials (35), which can be synchronized with presynaptic potentials generated by the TMS pulse to generate either synaptic potentiation or synaptic depression according to models of Hebbian plasticity [see also (36) for a discussion of this approach]. Considerable progress has been made in decoding cortical excitability states from the phase of brain oscillations to guide the induction of plasticity, which we describe in the next 2 sections. We start with studies in the motor cortex that were seminal for developing the EEG-informed TMS (EEG-TMS) technology and then move to studies in the prefrontal cortex, which is the established target for treating many disorders in psychiatry with rTMS. Figure 3 shows that different predominating endogenous rhythms are prevalent in these areas as temporal targets for EEG-TMS approaches. While it is the sensorimotor mu rhythm in the alpha frequency range in the motor cortex, it is a theta rhythm in the midline prefrontal cortex.

## Brain State–Dependent Stimulation and Closed-Loop Stimulation for Plasticity Induction in the Motor Cortex

We and others have demonstrated that the phase of the ongoing local sensorimotor mu rhythm, an approximately 10-Hz oscillation (cf. Figure 3), has a significant impact on MEP amplitude, an index of corticospinal excitability: TMS pulses delivered at the trough or ascending phase result, on average, in larger MEPs than otherwise identical TMS pulses targeting the positive peak or the falling phase (14,15,37, 38, 39, 40, 41). Therefore, the trough and ascending phase of the sensorimotor mu rhythm are high-excitability states of the corticospinal system. The EEG signal of the mu rhythm that displays the modulatory effect on corticospinal excitability originates from the postcentral gyrus, i.e., the somatosensory cortex, while EEG signals of the mu rhythm that originate from the precentral gyrus, i.e., the motor and premotor cortex, do not show this effect (42,43). This strongly suggests that the excitability of motor cortex output neurons is controlled by inputs from other areas that form the sensorimotor cortical network.

In addition to phase, power of the mu rhythm also impacts MEP amplitude, with high power at the time of the TMS pulse resulting in larger MEP amplitude (16,17,38,41). Moreover, mu phase and power interact, with the highest corticospinal excitability state occurring at the trough or ascending phase and high power and the lowest excitability occurring at the trough and low power (44), but this interaction was not replicated in one other study, where the effects of mu phase and power were independent (41). In contrast to corticospinal excitability as measured by MEP amplitude, short-interval intracortical inhibition, a paired-pulse TMS marker of GABAergic (gamma-aminobutyric acidergic) inhibition in the motor cortex (45), is not modulated by mu phase or power (38). Finally, the trough of the mu rhythm can also be demonstrated as a high-excitability state compared with the peak of the mu rhythm by larger TMS-evoked EEG responses (46). This is a relevant extension to the MEP findings, indicating the possibility that fluctuation of excitability states can also be measured by TMS-EEG responses, potentially even outside the motor cortex (47).

A hallmark of long-term synaptic plasticity induction as investigated in slice preparations is cooperativity. The induction of LTP will be facilitated if stimulation is paired with postsynaptic depolarization either by concurrent synaptic input or somatic current injection (48). Similarly, at the systems level of the human motor cortex, pairing depolarization by anodal transcranial direct current stimulation with rTMS enhances LTP-like plasticity as indicated by a lasting increase in MEP amplitude, while pairing hyperpolarizing cathodal transcranial direct current stimulation with rTMS switches LTP- to LTD-like plasticity (49). Therefore, the hypothesis was put forward that endogenous high-excitability states, such as the trough of the sensorimotor mu rhythm, may also enhance induction of LTP-like plasticity. To test this hypothesis requires a millisecond-precise real-time estimation of the phase of the ongoing mu rhythm to consistently trigger TMS pulses at a specified phase (15). Consistent with this hypothesis, 200 high-frequency (100–200 Hz) TMS triplets applied at the troughs of the ongoing mu rhythm result in an LTP-like increase in MEP amplitude, whereas the identical TMS triplets applied during the peaks or at random phases of the mu rhythm do not change MEP amplitude (15,50). Other TMS triplet frequencies (60 Hz, 666 Hz) are not effective at inducing LTP-like plasticity (50), an effect that is different from the 50-Hz triplets in conventional open-loop theta burst stimulation protocols but concordant with the natural occurrence of phase-amplitude coupling of high-gamma with slower oscillations in the human cortex (51). Conversely, the LTD-like effect of low-frequency rTMS is larger when TMS pulses are given during the corticospinal low-excitability state, i.e., the peak of the ongoing mu rhythm (52). Brain state–dependent stimulation based on the real-time determination of mu phase also has behavioral relevance. Offline motor skill learning is enhanced only by rTMS during troughs of the mu rhythm applied directly after skill practice but not by the same rTMS during peaks of the mu rhythm or sham stimulation (53).

Adaptive closed-loop brain state–dependent stimulation of the motor cortex for optimization of plasticity induction has not been realized yet to the best of our knowledge. Such approaches would require a second-order update algorithm that continuously compares the real output (e.g., MEP amplitude) with a reference and adjusts stimulation parameters to decrease the difference between real output and reference (cf. Figure 2). However, the feasibility of this approach has been demonstrated by automated determination of the optimal TMS site and orientation of the induced current to elicit MEPs of maximal amplitude in motor cortex mapping studies, using Bayesian optimization and a multichannel TMS transducer (54).

## Brain State–Dependent Stimulation in the PFC

How can we translate these findings demonstrating the feasibility of brain state–dependent TMS in the motor cortex to apply similar approaches in other brain areas? With regard to the treatment of MDD with rTMS, the main targets are left DLPFC and EEG studies that are aimed at identifying biomarkers of the pathophysiology of MDD that have implicated 8- to 12-Hz alpha oscillations (55), specifically an overactivity of left prefrontal versus right prefrontal alpha oscillation amplitude (56). Accordingly, conventional rTMS protocols for the treatment of MDD use a frequency of 10 Hz (1), with some studies adjusting the rTMS frequency to match the individual EEG alpha peak (57). The initial clinical pilot studies of brain state–dependent TMS of the left DLPFC in MDD have also targeted frontal EEG oscillations in the alpha band, using a similar approach to TMS of M1 triggered by sensorimotor alpha. The clinical feasibility of real-time alpha oscillation–synchronized TMS of the left DLPFC in the treatment of MDD has been demonstrated in 2 independent pilot studies (58,59) showing promising neurophysiological effects in the EEG-synchronized condition.

Recent developments in rTMS for MDD have investigated shorter stimulation protocols with a 5-Hz carrier frequency (i.e., theta burst stimulation) that have a similar therapeutic efficacy to 10-Hz rTMS (3,7). Regarding spatial targets, the dorsomedial PFC (DMPFC) has also been identified as a possible rTMS treatment target for MDD (60, 61, 62, 63, 64). The dominant brain rhythm at this location is the 4 to 7 Hz midfrontal theta oscillation (cf. Figure 3), which makes this a candidate for investigations into the potential of theta oscillation–synchronized therapeutic EEG-TMS of the DMPFC. The prefrontal theta rhythm has been implicated in neurocognitive processes, such as working memory (65,66) but also in mood control (67). In particular, the increase of prefrontal activity in the theta band after electroconvulsive therapy (68) and the increase in frontoparietal connectivity in the theta band after magnetic seizure therapy (69) have been found to be predictive of treatment response in patients with MDD.

Recent methodological advances have enabled the accurate extraction of EEG originating at the DMPFC using real-time EEG source localization based on beamforming and individual anatomical modeling (70). Real-time estimation of theta phase in the human prefrontal cortex from EEG signals poses specific challenges because the signal-to-noise ratio is low, and theta oscillations typically occur in periods (bursts) of <2 to 4 seconds (71), rendering phase prediction prone to errors, such as sudden changes in signal power and phase resets (72). However, a real-time phase-estimation accuracy in the range of ±55° can be achieved with individualized source-based spatial EEG filters rather than a standard Laplacian filter and by avoiding periods of low theta power and periods containing a phase reset (70). This accuracy is comparable to the one for mu phase detection over the motor cortex (15,42).

Applying this methodology to target theta oscillations originating from sources in the DMPFC, it was shown in healthy participants that 400 gamma-frequency (100 Hz) TMS triplets increased single-pulse TMS-induced prefrontal theta power and theta-gamma phase-amplitude coupling when the triplets targeted the trough of the ongoing theta oscillation (73). In contrast, prefrontal theta power decreased when gamma-triplet TMS targeted the theta rhythm peak, and no change occurred with random phase stimulation. In addition, gamma-triplet TMS at the trough of the theta rhythm improved reaction time in a working memory task, and this behavioral benefit was correlated with the magnitude of the increase in prefrontal theta power (73).

While speculative at this point in time because no clinical trials have been performed yet, we believe that brain state–dependent stimulation targeting midfrontal theta phase with EEG-TMS may increase therapeutic efficacy compared with conventional open-loop fixed stimulation protocols. These benefits will be achieved by more effectively increasing power or connectivity in the theta band, putative markers of a therapeutic response to brain stimulation in MDD.

## Current Challenges of Implementing Closed-Loop Therapeutic TMS in Psychiatry

The considerable engineering challenges of combining EEG and TMS have largely been solved (74), and the online execution of sophisticated real-time signal analysis algorithms has become possible on commodity computing hardware (15,42). Nevertheless, a number of significant barriers remain before a circuit-specific personalized closed-loop neuromodulation approach can become a viable treatment option for neuropsychiatric patients.

Considering the first-order trigger function of a personalized TMS therapy system, in some sense, brain state–dependent stimulation makes the situation worse because in addition to optimizing the stimulation parameter space (coil location, stimulus intensity, pulse pattern, etc.), we now also need to decide which EEG-defined brain state to synchronize the stimuli to. In practice, we are limited to oscillatory measures with a sufficient signal-to-noise ratio to enable reliable single-trial estimation (72), such as midfrontal theta, occipital alpha, and sensorimotor alpha (Figure 3). After having selected an oscillation of interest, we also need to make a decision about the specific phase to synchronize to, with the goal of maximizing therapeutic efficacy, and it is not self-evident that this should be the trough (14). The neuroplastic effects of synchronizing TMS with different phases of different oscillations are difficult to predict and would need to be determined in time-consuming studies, even if such studies did not individually adjust the EEG montage and target phase. Beyond the targeting phase and/or power of an ongoing oscillation, more complex EEG markers such as combined spatiotemporal EEG signatures (19), coherence-based connectivity states, or distributed oscillatory states (75) may need to be considered.

Once a relevant oscillation and its specific phase have been determined, the target brain state needs to be estimated with sufficient reliability to achieve synchronization in the presence of artifacts and noise. EEG analysis traditionally makes extensive use of averaging to reduce the influence of noise, but of course this is not possible when using a real-time instantaneous state estimation system (72). Even in an EEG signal that is free of artifacts, isolating the relevant oscillation from the spatially mixed sensor signal is challenging (76), especially when such mixing can result in spurious higher-frequency oscillations, as in the case of beta oscillations resulting from mixed nonsinusoidal alpha sources (77). Therefore, it is not enough to simply extract a real-time measure of brain state; it is also necessary to simultaneously estimate the reliability of that measure, at that time, in the presence of noise (e.g., blink, muscle, electrode) and other brain sources. In summary, the real-time algorithm also needs to detect different kinds of artifacts and, as a result, the determination of when not to stimulate, due to excessive estimator error variance, may be more complex and take more computational resources than the target brain state detection itself.

Considering the second-order update function of a future closed-loop therapy system, at first this seems to complicate the overall situation further. However, the degrees of freedom available to the trigger function (at which location, at what intensity, with which pattern, in response to which oscillation, and at what phase) are precisely the domain that the update function operates on. Therefore, it may serve as a solution to the problem of finding the individual stimulation parameters, acting as an optimization process that minimizes the difference between the desired and the actual situation. It is not obvious how to extract this information from the EEG signal while the desired long-term neuroplastic effect is still taking place. In suprathreshold TMS of the primary motor cortex, the MEP amplitude is a readily available single-trial marker of corticospinal excitability that can be quantified easily. The lack of a similarly accessible single-trial marker of cortical excitability, outside of the motor system, is perhaps the most significant barrier preventing the translation of the approach used in the motor system to the frontal cortex, although recent efforts have made some progress toward this goal (78, 79, 80, 81). Therapeutic closed-loop stimulation for psychiatric disorders would monitor a TMS-EEG marker that reflects the activity of the dysfunctional brain networks and would continuously optimize the configuration of the intervention within a treatment session and from one session to the next.

## Outlook

In light of the preceding challenges, realizing the full version of closed-loop therapeutic brain stimulation, as understood in this analysis, may not seem immediately achievable. The pertinent question is how much more effective a given brain intervention would be if it were applied in an optimally personalized way and which neurophysiological research questions do we need to address to get there. Can the recently reported dramatic effects of invasive closed-loop neurostimulation in an *n*-of-1 study in a patient with severe therapy-resistant MDD (82) be replicated with EEG-TMS? That study identified a personalized, symptom-specific biomarker (bilateral amygdala gamma power) and a treatment location (right ventral capsule/ventral striatum) where stimulation improved symptoms using multiday intracranial electrophysiology and focal electrical stimulation prior to implantation of a sensing and stimulating-responsive DBS device for closed-loop stimulation (82). A similar scenario may be possible for EEG-TMS with a multisession calibration phase to identify an individual EEG-based biomarker associated with high symptom severity (81) and a site where and a timing when rTMS is particularly effective in modifying the biomarker and symptom severity. Informed by this biomarker identification, the application of longer-term therapeutic closed-loop stimulation should follow.

There is an urgent medical need to further develop these kinds of therapies to better help the 1 billion people globally who, in any given year, experience a debilitating neuropsychiatric brain disorder, as well as to reduce the stigmatizing side effects of pharmacotherapy. We acknowledge that barriers remain before noninvasive, personalized circuit-based therapies can enter routine clinical practice, but we expect that recent systematic progress addressing the relevant neurophysiological and clinical questions will continue steadily. More specifically, we consider the identification of single-trial TMS-EEG markers of frontal network excitability to be of critical importance, not only to determine whether a given coil position and intensity functionally engages the relevant target network, but also as a biomarker for plastic change. We also consider it necessary to evaluate the neuromodulatory effects of rTMS to different target locations and synchronized to different phases of a target oscillation, for which we propose midfrontal theta as a promising candidate. The remaining engineering challenges that need to be solved to enable widespread use of this approach are relatively straightforward. From a technical point of view, a small number of dry EEG sensors will likely be sufficient, with modest signal-processing requirements that can be used in conjunction with existing TMS devices. Therefore, the development of a significantly more effective next generation of personalized EEG-TMS therapy systems seems clinically feasible, and we believe that closed-loop stimulation will play a decisive role in realizing the full therapeutic potential of this approach.
